# 

**Published:** 1895-06

**Authors:** 


THE American Dental Association holds its next annual meeting at Asbury Park, N. J., Tuesday, August 6th, 1895. I am requested by the President, Dr. J. Y. Crawford, to give notice that it is the privilege and duty of each State and Local Society to appoint and send delegates to the American Association.

Each State and Local Society which has adopted substantially the same Code of Ethics as that governing the conduct of members of the American Dental Association is entitled to one representative for every five members and fractional part thereof.

Blank certificates for delegates may be had on application to Corresponding Secretary.

Many questions of interest will come up for discussion at this meeting, and every Dental Society in the United States should be fully represented in this, our National Convention.

EMMA EAMES CHASE,
Corresponding Secretary,
American Dental Association.

ST. LOUIS, MO. April 27th, 1895.

### East Tennessee Dental Association.

The twenty-eighth annual meeting of this Association will be held June 11th to 13th inclusive, at Harriman Tennessee. A very interesting programme has been prepared in which, among other subjects there is Alveolar Abscess. Crown and Bridge-work. Dental Medicine. Plastic Fillings. Gold Fillings. Diseases of the Oral Cavity and Treatment. How to Build up a Practice and how to lose it. Wednesday morning will be devoted to clinics.

A general and cordial invitation is extended to all who can possibly attend. The usual reduction in railroad rates will be given.

The citizens of "Happy Harriman" will entertain the East Tennessee Dental Association in a manner which would do credit to an older town than five years. A banquet on night of 13th, an excursion on 14th to State Mines on the H. C. & I. R. R. The excursion committee have gotten up a splendid programme, and taking all things together, the twenty-eighth annual meeting should eclipse all previous meetings held by this Association. Let all who can possibly do so, Go!

Most Respectfully Yours,
GEO. C. BRAUSE, *Secretary.*

### National Association of Dental Faculties.

The annual meeting of this body will be held at Asbury Park, N. J., on Saturday, August 3d, at 10 o'clock A. M. It is very desirable that all colleges having membership be promptly present at that hour, as much important business will be before the association, and the time allotted is usually short for the work to be done.

The Executive Committee of the association will meet on Friday previous at 10 o'clock, at the same place. All business for that committee should, so far as possible be in their hands before the meeting, in order that there may be no delay.

J. TAFT,
Chair. Ex. Com.

### Tri-State Meeting.

The State Dental Associations of Ohio, Indiana and Michigan will hold a union meeting in the City of Detroit on the 18th to the 20th of June, inclusive, 1895.

It is very desirous that there be a full attendance on the part of each of these States as preparations have been made for a large number. The occasion will be one not only of professional interest, but of social enjoyment as well. Indiana and Ohio will be the guests of Michigan in a social way, and if the guests are equal in number to the hospitality of the host it will be a rousing time.

The Meetings and Clinics will be held in the Dental Department of the Detroit College of Medicine and Surgery where ample accommodation for all the work will be found.

The head-quarters will be at the Russell House. The Normandy is conveniently situated and will afford excellent accommodation.

Railroad rates have been secured at the usual one and one-third rate, within the Central Traffic Association territory, this embraces all the states here named.

To make this arrangement available certificates must be taken where the tickets are purchased and these certificates must be signed at the meeting by Dr. G. E. Hunt.

Only a partial program has as yet appeared but we are warranted in promising a splendid list of subjects, for both papers and clinics. In both these directions there will be much presented of intense interest; so come and bring your largest intellectual pocket with you.

### CONTENTS.

COMMUNICATIONS.

### THE DENTAL REGISTER,

**A MONTHLY MAGAZINE DEVOTED TO THE INTERESTS OF THE**

### DENTAL PROFESSION.

*Terms Reduced to* $2.00 *Per Year. Single Copies,* 25 *Cents.*

**EDITED BY PROF. J. TAFT, D.D.S.**

—AND—

**Published by SAM'L A. CROCKER & CO., Ohio Dental and Surgical Depot.**

The volume commences in January and closes in December.

The REGISTER being issued monthly is always fresh, therefore Indispensable to the practitioner who desires to keep posted up with the new inventions and improvements which are daily developed. Neither expense nor effort will be spared to give value and variety to its contents Articles will be illustrated with well executed engravings whenever they will add to the interest or assist to explanation.

Its extensive circulation and frequency of issue make it one of the BEST DENTAL ADVERTISING MEDIUMS in the United States.

All subscriptions must begin with January or July.

Local or special notices, five lines or less, will be inserted for $3 each insertion; over five lines, 50 cts. per line.

All advertising bills payable in advance, or at furthest, after the issue of the first number containing the advertisement. All transient advertisements to be paid for in advance.

☞CONTRIBUTIONS SOLICITED.

Exclusively Editorial Communications should be addressed to the Editor.

The latest number of the REGISTER may be ordered with or without other goods any time, and all letters should be addressed to

